# Reprioritizing Genetic Associations in Hit Regions Using LASSO-Based Resample Model Averaging

**DOI:** 10.1002/gepi.21639

**Published:** 2012-04-30

**Authors:** William Valdar, Jeremy Sabourin, Andrew Nobel, Christopher C Holmes

**Affiliations:** 1 Department of Genetics, and Lineberger Comprehensive Cancer Center, University of North Carolina at Chapel HillChapel Hill, North Carolina; 2 Department of Statistics and Operations Research, University of North Carolina at Chapel HillChapel Hill, North Carolina; 3 Department of StatisticsOxford, United Kingdom

**Keywords:** GWAS, case-control, genotype imputation, model averaging, LASSO, Stability Selection

## Abstract

Significance testing one SNP at a time has proven useful for identifying genomic regions that harbor variants affecting human disease. But after an initial genome scan has identified a “hit region” of association, single-locus approaches can falter. Local linkage disequilibrium (LD) can make both the number of underlying true signals and their identities ambiguous. Simultaneous modeling of multiple loci should help. However, it is typically applied ad hoc: conditioning on the top SNPs, with limited exploration of the model space and no assessment of how sensitive model choice was to sampling variability. Formal alternatives exist but are seldom used. Bayesian variable selection is coherent but requires specifying a full joint model, including priors on parameters and the model space. Penalized regression methods (e.g., LASSO) appear promising but require calibration, and, once calibrated, lead to a choice of SNPs that can be misleadingly decisive. We present a general method for characterizing uncertainty in model choice that is tailored to reprioritizing SNPs within a hit region under strong LD. Our method, LASSO local automatic regularization resample model averaging (LLARRMA), combines LASSO shrinkage with resample model averaging and multiple imputation, estimating for each SNP the probability that it would be included in a multi-SNP model in alternative realizations of the data. We apply LLARRMA to simulations based on case-control genome-wide association studies data, and find that when there are several causal loci and strong LD, LLARRMA identifies a set of candidates that is enriched for true signals relative to single locus analysis and to the recently proposed method of Stability Selection. Genet. Epidemiol. 36:451–462, 2012. © 2012 Wiley Periodicals, Inc.

## INTRODUCTION

Single locus regression has become a staple tool of human genome-wide association studies (GWAS; WTCCC [2007]). Despite the fact that it simplistically reduces the often complex genetic architecture of a phenotype down to effects at an individual single nucleotide polymorphism (SNP) (or other localized variant), it has proved powerful in identifying major genetic determinants and predictors of disease susceptibility [Cantor et al., 2010]. Many would acknowledge that simultaneous modeling of all loci potentially yields fairer estimates of genetic effect, more stable phenotypic predictions, and better characterization of between-locus confounding [Hoggart et al., 2008; Lee et al., 2008]. However, such multiple locus approaches are at present seldom used. This could be because they are considered impractical, potentially hard for readers to understand, or, with some theoretical support [Fan and Lv, 2008], unnecessary in an initial genome scan. Certainly, much of the genome-wide confounding that explicit multiple locus modeling would hope to resolve is efficiently, if bluntly, dealt with by the addition of regression covariates correcting for higher order geometric relationships in the data [Price et al., 2010] or probabilistically inferred strata [Pritchard et al., 2000].

Nonetheless, once initial genome scans have been performed and “hit regions” of association identified, shortcomings of a single-locus approach become apparent. Local patterns of linkage disequilibrium (LD) in such hit regions can make ambiguous both the number of underlying true signals and the identity of the loci that most directly give rise to them [e.g., Strange et al., 2010]. Statistical analysis after this point is often ad hoc. It typically involves fitting further regressions that condition on “top” loci that appear most strongly associated in order to rule out neighbors or rule in suspicions of an independent second signal [Barratt et al., 2004; Udea et al., 2003]. This is followed by more interpretive analysis based on annotation as a prelude to, for example, investigation at the bench. In ad hoc conditioning, rarely is there formal consideration of the fact that the association of the top locus is often insignificantly different from that of its correlated neighbors, and that whereas its association with the phenotype is probably stable to sampling error, its superiority in association over its neighbors is probably not. This inherent instability of the relative strengths of association between confounding loci makes such strategies high risk: a slightly different sampling of individuals could demote the conditioning locus, result in an alternative conditioning locus being chosen, and potentially lead to altered conclusions. This approach becomes yet more unstable when some of the loci are themselves known with varying certainty, their genotypes having been partially or wholly imputed [Zheng et al., 2011], such that weakness of association is now also a function of imputation uncertainty unrelated to the phenotype [e.g., Servin and Stephens, 2007].

There is thus great value in developing principled approaches to discriminate true from false signals in hit regions. Joint modeling of all loci through multiple regression seems attractive because it accounts for the LD of the data [Balding, 2006]. Standard regression is unsuitable for this purpose, however, because even when the number of considered loci *p* is much fewer than the number of individuals *n*, LD creates multicollinearity that derails meaningful estimation of locus effects. Stepwise multiple regression techniques [Cordell and Clayton, 2002] formalize the ad hoc conditioning approach but also inherit its weaknesses: model selection choosing a single set of active loci typically provides no indication about how sensitive that choice was to, for example, sampling variability, making it a statistic that is opaque at best and misleading at worst. Bayesian approaches offer a coherent perspective by formally accounting for uncertainty in model choice, effect estimation, and imputation uncertainty [Stephens and Balding, 2009]. Nonetheless, these are often highly computationally intensive, and require formal statements of prior belief relating to the number of causal variants and their effects that analysts may feel unprepared or unwilling to specify.

Penalized regression models can provide an alternative that does not require a commitment to Bayesian learning. Placing a penalty on the size of coefficients in the multiple regression leads to moderated estimates of coefficient effects, allowing their stable estimation even when many predictors are in the model. In particular, the LASSO [Tibshirani, 1996], which penalizes increases in the absolute value of each coefficient subject to a penalty parameter λ, results in some effects being shrunk to exactly zero. The result is a “sparse” model in which only a subset of effects are active. Increasing the level of penalization leads to greater sparsity, effectively making λ a continuous model selection parameter. Recent advances in fitting LASSO-type models have made them more practical for analysis of large-scale genetic data [e.g., Wu et al., 2009]. Nonetheless, as a tool for modeling effects at multiple loci, the LASSO leaves important questions unanswered. One problem is how to select λ. This is typically approached through criteria-based evaluation methods [Wu et al., 2009; Zhou et al., 2010], such as AIC and BIC, empirical measures of predictive accuracy (such as cross-validation [Friedman et al., 2010]), or criteria aiming to control type I error (such as permutation [Ayers and Cordell, 2010]). Another problem is, given λ, how to characterize uncertainty in model choice. Although LASSO moderates estimated effects through shrinkage, it is no better than stepwise methods in that it ultimately selects a single model (or single “path” of models, when λ is varied), and thus states with absolute confidence a statistic that could in fact be highly sensitive to the sampling of observations.

An intuitive way to characterize variability of model choice is to estimate for each locus a model inclusion probability (MIP). A Bayesian approach would formulate this as a posterior probability that conditions on both the observed data and prior uncertainty in model choice. The Bayesian MIP embodies a statement about whether the researcher should believe the locus is included in the true model. A frequentist alternative is to formulate the MIP as the probability a locus would be included in a sparse model under an alternative realization of the data. This frequentist MIP is thus a statement about the expected long-run behavior of the model selection procedure. Valdar et al. [2009] proposed an approach that applied forward selection of genetic loci to resamples of the data and defined the resample MIP (RMIP) as the proportion of resampled datasets for which a locus was selected. This resample model averaging (RMA) approach used either bootstrapping (i.e., “bagging”) or subsampling (i.e., “subagging”), and followed an earlier application to genome-wide association in Valdar et al. [2006] and work on general aggregation methods by Breiman [1996] and Bühlmann and Yu [2001] (cf. parallel applied work by Austin and Tu [2004] and Hoh et al. [2000]). Independently, Meinshausen and Bühlmann [2010] proposed “Stability Selection” (SS) that powerfully combines subagging with LASSO shrinkage to produce a set of frequentist MIPs at each specified λ. Recently, Alexander and Lange [2011] adapted this method with limited success to whole-genome association.

Herein, we propose a statistical method for reprioritizing genetic associations in a hit region of a human GWAS based on case-control data that exploits and extends the resample aggregation techniques developed in Valdar et al. [2009] and Meinshausen and Bühlmann [2010]. We demonstrate a principled approach, LASSO local automatic regularization resample model averaging (LLARRMA), that characterizes sensitivity of locus choice to sampling variability and uncertainty due to missing genotype data, and that provides LASSO shrinkage automatically regularized through either predictive- or discovery-based criteria. We show that when multiple correlated SNPs are present in a hit region that has been identified by standard single-locus regression, LLARRMA produces a reprioritization that is enriched for true signals.

## METHODS

We start by considering a standard logistic regression to estimate the effects of *m* SNPs in a hit region on a case-control outcome in *n* individuals, and then describe statistical approaches to identify a subset 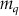 of SNPs that represent true signals. Herein, we define a “true signal” as the SNP that most strongly tags an underlying causal variant, a “background” SNP as an SNP that is not a true signal, and an optimal analysis as one that distinguishes true signals from background SNPs in the hit region. We assume that the hit region has been previously identified by an initial genome-wide screen using, for example, single-locus regression, that many of the *m* SNPs may be in high LD, and that 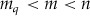. Let 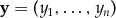 be an *n*-vector of the dichotomous response with each of the *n*_1_ cases coded by 1 and the *n*_0_ controls coded by 0, let 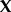 be an 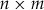 matrix of SNP genotypes, where SNPs are coded to reflect additive-only effects as 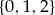 for unphased genotypes 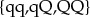, and let 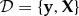 and 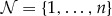. Logistic regression models the case-control status of individual *i* as if sampled from 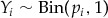, where *i*’s propensity 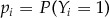 is determined by a linear function of the *m* SNP predictors


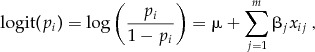
(1)

where 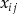 is the value of the *j*th SNP for the *i*th individual and the 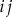
th element of the column-centered design matrix 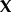, μ is the intercept, and 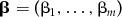 are the effects of the *m* predictors.

We assume that only a subset of the *m* SNPs are true signals, and define a corresponding vector of 0–1 inclusions 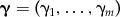 such that 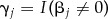. A common way to infer 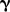, and to thereby estimate the identity of the true signal, is to use a model selection procedure that maximizes some criterion of fit. This returns a binary vector 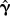, a hard estimate declaring which SNPs belong to the model. Although superficially attractive, 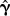 has limited interpretability because it provides no information about how sensitive the selection could have been to finite sampling. That is, whether 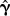 would be expected to vary dramatically when applied to alternative samples from the same population. Moreover, although many selection procedures guarantee that they will deliver the correct result in an infinite sample (i.e., are consistent), this offers little reassurance when the sample is finite, and suggests that the returned statistic 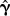 could have high variance.

### LLARRMA

#### Resample Model Averaging

We seek to estimate 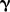 in a way that incorporates uncertainty in model choice arising through, for example, potential variability of the selected set due to finite sampling. To do this we use RMA [Valdar et al., 2009], applying a model selection procedure to repeated resamples of the data, and basing subsequent inference on the aggregate of those results. Rather than obtaining a binary estimate of each 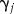, we instead seek to estimate its expectation 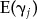 over resamples, hoping to approximate its expectation over samples from the population. We start by drawing subsamples 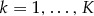 with subsampling proportion 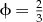, such that each subsample comprises data 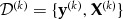 on 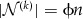 individuals 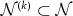. Each subsample is produced by drawing 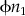 individuals at random without replacement from the *n*_1_ cases, and 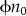 individuals at random without replacement from the *n*_0_ controls. For each subsample *k*, we perform a fixed model selection procedure to estimate 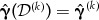, the *m*-length binary vector of inclusions based on the *k*th subsample. Applying this to all subsamples gives the 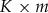 matrix 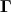, where 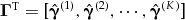. The expected proportion of times that the *j*th predictor is included in the model is given by its RMA estimate


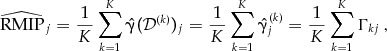
(2)

which we refer to as its RMIP.

#### Selection Within a Subsample Using the Lasso

To select SNPs within the *k*th subsample, we use LASSO penalized regression [Tibshirani, 1996]. This estimates 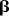 for subsample *k* as


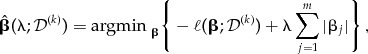
(3)

where 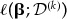 is the log-likelihood of 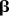 for data 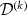, and λ is a penalty parameter. The LASSO estimate 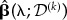 easily translates into an estimate of the inclusions 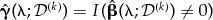. Nonetheless, to arrive at a single estimate of 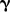, as required for model averaging, we must devise a suitable criterion for choosing the penalty λ. We propose two alternatives, both of which identify a value 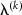 specific to subsample *k* (i.e., local): complement deviance selection and permutation selection.

#### Predictive-Based Choice of 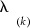: Complement Deviance Selection

The complement deviance criterion seeks a model that would perform well in out-of-sample prediction. After estimating 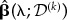 over a grid of λ to calculate the LASSO path, this criterion finds the value of λ that minimizes the deviance of the complement of subsample *k*, i.e.,


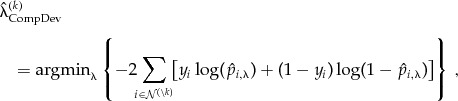


where 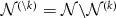 is the set of 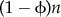 individuals not selected for subsample *k*, and 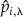 is the predicted probability of 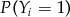 based upon 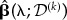 applied to the design matrix of the complement subsample 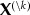.

#### Discovery-Based Choice of 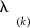: Permutation Selection

The permutation selection criterion is a modified version of that proposed by Ayers and Cordell [2010] and seeks a conservative model that would tend to include no SNPs under permutation of the response. Given a subsample *k*, we estimate for a given permutation of the response 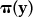 the smallest penalty required to zero out all predictors, i.e.,


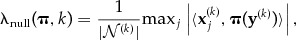


where 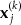 is the *j*th column of the subsampled and mean-centered design matrix 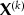, and 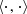 denotes the inner product of its two arguments. Calculating this for each of *S* permutations 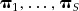, we estimate the permutation selection λ for subsample *k* as


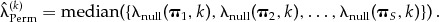
(4)

Ayers and Cordell [2010] apply a similar criterion when analyzing complete datasets, with the difference that they estimate 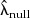 as the maximum of 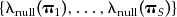 for 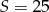. We prefer not to do this because the maximum is relatively unstable for 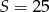, and is undesirable for larger *S* because it potentially allows 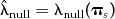 where 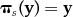. In contrast, when using the median (Equation 4) the accuracy of 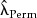 increases with *S*, although we find that in simulations 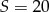 is adequate.

#### Incorporating Uncertainty Due to Missing Genotypes: Hard, Dosage, and Multiple Imputation

SNP data within a hit region will often include combinations of markers and individuals for which the genotype is unknown or uncertain. To avoid a potentially wasteful complete cases analysis, it is common to impute the missing genotypes using a program such as MACH [Li et al., 2010], IMPUTE [Howie et al., 2009], or fastPHASE [Scheet and Stephens, 2006], and analyze the partly imputed data as if it were fully observed. Imputation methods are typically based on reconstruction and phasing of inferred haplotypes. Dividing the SNP matrix 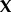 into missing and observed elements 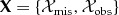, methods such as fastPHASE [Scheet and Stephens, 2006] model the joint distribution 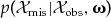, where 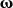 includes additional information used in the imputation (e.g., priors). Most GWAS, however, do not use this joint distribution directly. Rather, they replace 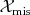 with a point estimate 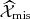, each element of which is constructed from its marginal distributions. Specifically, 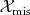 is replaced by either the “dosage,” 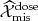, with elements defined as the expectation of the allele count 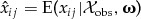; or a “hard” imputation, 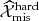, with elements imputed as their maximum a posteriori genotype


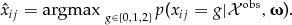


The simplest approach to modeling missing genotypes within LLARRMA is first to estimate 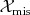 as either 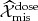 or 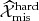 and then subsample 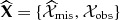 as if it were complete. This plug-in approach underestimates variability because it fails to incorporate uncertainty about the imputation. Zheng et al. [2011] show that doing this when modeling effects at single loci reduces power by a negligible amount when the imputation accuracy is reasonably high. Nonetheless, ignoring imputation uncertainty could be more problematic in multiple-locus settings, if, for example, the posterior distribution of haplotypes 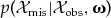 differs substantially from joint distribution implied by the product of marginal posteriors 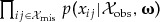 [e.g., Servin and Stephens, 2007]. A natural way to incorporate imputation uncertainty into our resampling framework is through multiple imputation [Little and Rubin, 2002]. At each iteration *k*, we sample a new 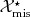 from its posterior 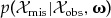, subsample the resulting 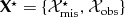 to give 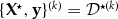, and then calculate RMIPs using 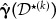 in place of 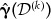 in Equation 2. The resulting RMIPs incorporate additional variability because each subsample now includes a potentially different imputation of missing genotypes. We implement hard, dosage, and multiple imputation using posterior draws from fastPHASE (making use of the -s option).

### COMPETING METHODS

LLARRMA calculates a score (an RMIP) for each SNP in a case-control study. We compare the ability of those scores to discriminate true signals from background with the SNP scores calculated by two alternatives: the traditional GWAS approach of single-locus regression, and the LASSO-based subsample model averaging method stability selection (SS) recently proposed in a more general context by Meinshausen and Bühlmann [2010].

#### Single Locus Regression

We perform single locus regression with logistic regression as used in, for example, PLINK [Purcell et al., 2007]. For each SNP, we fit a single-predictor version of Equation 1 and score its 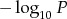 (log*P*), where *P* is the *P*-value from a likelihood ratio test against an intercept-only model.

#### Stability Selection

**Fig. 1:**
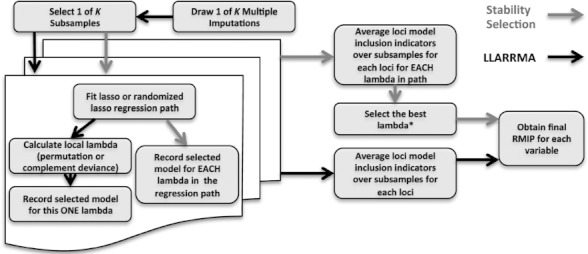
A comparison of LLARRMA and Stability Selection.

SS differs from LLARRMA in two main respects (see Fig. 1). First, whereas LLARRMA selects variables within each subsample using a local (i.e., subsample-specific) penalty 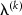, SS uses a single global penalty λ applied to all *K* subsamples. Second, whereas LLARRMA chooses each 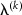 automatically, SS leaves its global λ as a free parameter. In SS, the RMIP (referred to as the “selection probability” in Meinshausen and Bühlmann [2010]) is thus left as a function of λ,


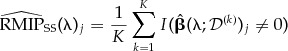
(5)

giving rise to a sequence of RMIPs (a “stability path”) for each locus *j*. Meinshausen and Bühlmann [2010] provide little guidance for choosing λ. As a choice of λ is required to produce a unique RMIP and thereby ensure meaningful comparison with LLARRMA, we select λ to produce the stiffest possible competition: as the value that maximizes the criterion used for comparing methods. Specifically, given a criterion of success 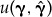 comparing truth 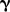 with guess 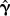, we define


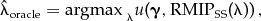


where “oracle” reflects the fact that choosing this unfairly advantageous value requires foreknowledge of 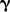. We consider SS with the oracle property defined by setting *u* to be the initial area under the curve (AUC) (described below).

### ROC-BASED EVALUATION

We assess the performance of LLARRMA and its competitors by simulation, examining the ability of each to discriminate true signals from background in simulated case-control studies. Performance is evaluated formally using receiver operator characteristic (ROC) curves. ROC curve methodology can vary between studies [Krzanowski and Hand, 2009], so we describe ours in full. A given simulation study comprises a set of simulation trials 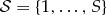. In each trial *s*, a given method is presented with *m* SNPs of which 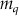 will be a true signal. That method calculates a single score for each SNP (an RMIP or log*P*). For a given threshold *t*, define 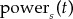 as the proportion of 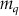 true signal SNPs scoring 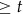 (i.e., the power to detect), and the false-positive rate 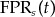 as the proportion of the 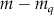 background SNPs scoring 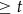 (i.e., the false positive rate; FPR). We define the area under curve in trial *s* for FPRs between *a* and *b* as 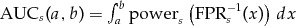, where 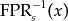 returns the threshold *t* at which the FPR is *x*, and the integration is approximated using the trapezium rule. For a given method and set of simulations 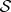, we define the estimated AUC between FPR *a* and *b* as 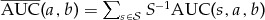, and assume this estimate to be approximately normally distributed with variance 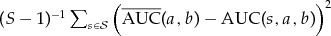. We define the “initial ROC” as the ROC curve in the range FPR ∈ [0, 0.05], and the “initial AUC” as 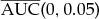; the “full ROC” is where FPR ∈ [0, 1] and the “full AUC” is 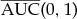. When plotting ROC curves for each method, we use threshold averaging [Fawcett, 2006], varying *t* over its range ([0, 1] for RMIPs; [0, ∞) for log*P*) and at each *t* plotting *x* and *y* coordinates 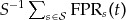 and 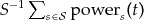, respectively.

### SIMULATION STUDY 1: FIVE LOCI IN CANCER DATA

We obtained genotype data from phase 1 of a case-control GWAS for colorectal cancer from collaborators at the Wellcome Trust Centre for Human Genetics, University of Oxford. Two forms of the data are used here. The “cancer data” comprise complete genotype information on 1,493 subjects for 183 SNPs covering a hit region previously identified on 18q21. The cancer data are a subset of the “full cancer data,” which comprises incomplete genotype information on 1,859 subjects for the hit region.

#### Generating Missing Genotypes

To assess the sensitivity of the compared methods to alternative strategies for modeling missing genotypes, we generate incomplete versions of the cancer data by deleting genotypes according to a random missingness algorithm. The missingness algorithm is based on empirical modeling of the pattern of missing data in the full cancer data. The full cancer data genotypes contained 854 missing genotypes (∼0.25%). We observed that the proportion of missing genotypes varied considerably from SNP to SNP, but that missingness across individuals was consistent with a random allocation. To generate each incomplete dataset, we therefore do the following. First, for each SNP *j*, we assign a missingness proportion 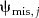 generated as a random draw 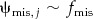, where *f*_mis_ is an empirical density based on the histogram of missingness proportions of SNPs in the full cancer data. Second, we select a subset of 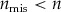 individuals eligible to receive missing genotypes. Third, at each SNP *j* we delete 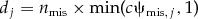 marker genotypes at random from the *n*_mis_ individuals, where *c* is chosen such that the overall proportion of missing data is fixed value 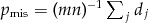. To generate a more conservative level of missingness while ensuring at least 10% of individuals had complete data, we set 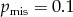 and 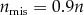.

#### Simulating Phenotypes

Phenotypes are simulated based on a binomial draw from the logistic model in Equation 1. Given a set of SNPs representing true signals, with genotypes 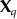 and effects 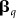, we first calculate the intercept necessary for an expected 50/50 ratio of cases to controls as 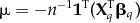, calculate individual propensities as 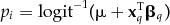, and then draw phenotypes as 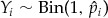.

#### Placing Causal Loci

**Fig. 2:**
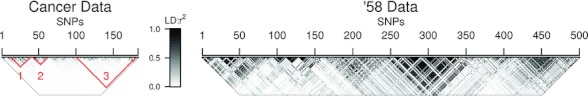
LD structure of the two genotype datasets used in the simulations. Shading indicates pairwise LD between SNPs, ranging from 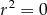 (white) to 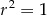 (black). Red highlighting shows blocks where true signals were placed in simulation studies 1A and 1B.

To ensure a degree of confounding correlation between loci, we choose five true signal SNPs at random but in a restricted manner from the LD blocks shown in Figure 2. Specifically, in each simulation trial, two SNPs are chosen from block 1 at random but subject to correlation 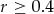, two SNPs are from block 2, also subject to 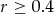, and one SNP is randomly chosen from block 3.

#### Simulation 1A: Moderate Effects

To aid an initial illustrative comparison between methods, our first study on the cancer data simulates a relatively constant effects structure. In each simulation trial, we assign a permutation of the effects (on the odds scale) 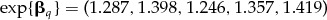 to the selected five SNPs.

#### Simulation 1B: Small Effects

Providing a more challenging and variable set of causal targets, our second study on the cancer data randomly chooses true signal SNPs as in 1A but draws each element 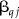 of effects 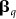 independently as 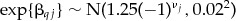 with 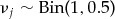. The resulting effects are comparable to the small effects estimated in many GWAS [Manolio et al., 2009].

### Simulation Study 2: one to Seven Loci in ‘58 Data

The “‘58” data are a complete-genotypes subset of data collected during the human GWAS for seven diseases described in WTCCC [2007]. It comprises genotypes for 2,199 subjects on 500 SNPs in the region 39.063723–40.985321 Mb on chromosome 22, this region being chosen by us as a contiguous run of markers that exhibits a mixture of high and low LD (Fig. 2). To assess the how the number of true signals affects the relative utility of modeling single vs. multiple loci, we evaluated methods in seven distinct simulation substudies, simulating 1, …, 7 true signals, respectively. In each simulated trial of each substudy, the set of true signals is chosen at entirely random from the 500 SNPs and the SNP effects are generated as in simulation 1B above.

### COMPUTATION

Genotype imputation was performed using fastPHASE [Scheet and Stephens, 2006]. All other analyses were performed in R [R Development Core Team, 2011], with the *glmnet* package [Friedman et al., 2010] used for fitting LASSO models. On a 2.4 Ghz MacBook Pro with 4 Gb RAM, on average 100 subsamples on the cancer data take the following times: LLARRMA with permutation selection, 39.8 sec (SD = 2.6 sec); LLARRMA with complement deviance selection, 389.2 sec (SD = 64.6 sec); SS, 305.7 sec (SD = 48.5 s). Use of multiple/hard/dosage imputed data incurs negligible extra computation, assuming the imputation itself has been done in advance.

## RESULTS

### SIMULATION STUDY 1A: MODERATE LD, MODERATE EFFECTS

We simulated 1,000 case-control datasets based on the cancer data (see Methods and Fig. 2). Each simulated dataset had approximately balanced cases and controls, with individuals’ outcomes influenced by five SNPs of moderate effect (odds ratios 1.246–1.419) out of 183 SNPs in total, and existed in both a complete form, referred to as the “complete” dataset, and an incomplete form, in which some genotype values were set to be missing. The incomplete form was available in three alternative imputations: a “hard” imputation, a “dosage” imputation, and an ensemble of 100 sampled imputations that constituted a single “multiple” imputation set (these imputations being generated by fastPHASE [Scheet and Stephens, 2006]). At each simulation, we tested four different analysis methods that each produced a score per SNP. Our subsequent comparisons of those methods were based on how well their scores discriminated the five SNPs that represented true signals from the 178 that did not. The four methods examined were (*short names in parentheses*): single SNP logistic regression (*single-locus regression*); LLARRMA using permutation selection (*permutation selection*); LLARRMA using complement deviance selection (*complement deviance*); SS using oracle penalization (*oracle* *SS*). All methods were applied to the complete, hard imputation, and dosage imputation versions of each simulated dataset; resample-based methods (i.e., all except single-locus regression), which were set to use 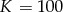 subsamples, were also applied to the multiple imputation set.

#### An Example Simulation

**Fig. 3:**
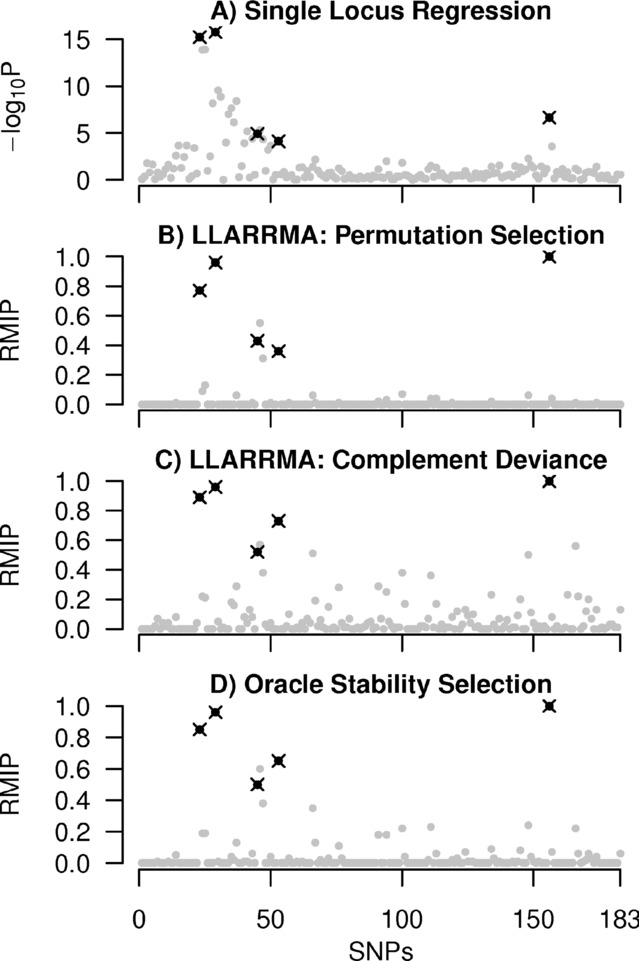
Results of four methods applied to an example case-control dataset from simulation study 1A. Plots show SNP score (log*P* or RMIP) against SNP location in the cancer data, with true signal SNPs in black and background SNPs in gray.

Figure 3 plots SNP location against SNP-score for each method in an example simulation applied to complete data. True signal SNPs are plotted as black crosses and the remaining (background) SNPs as gray dots. In single-locus regression (Fig. 3A), SNPs are scored as 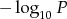 (log*P*; see Methods). Although the true signals between 1 and 50 tend to attract higher scores, so do many of the backgrounds SNPs between 1 and 60, giving rise to a cloud of association that is characteristic of many hit regions in real GWAS. The remaining methods (3 B–D) report inclusion probabilities (RMIPs) for each SNP. These describe a frequentist probability that each SNP would be included in a sparse model that seeks to estimate the joint effects of multiple SNPs. Because SNPs compete with each other for inclusion in these methods, the resulting scores more clearly differentiate those SNPs. In this example, that increased sparsity coincides with the set of higher scored loci being more enriched for true signals than is the case with single-locus regression.

#### Results From 1,000 Simulations

**Fig. 4:**
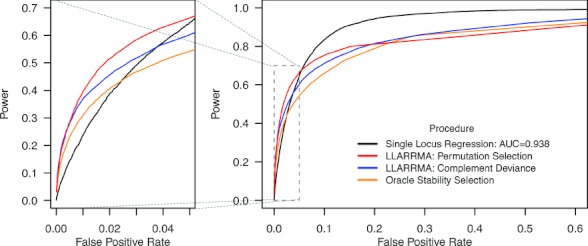
Receiver operator characteristic (ROC) curves for simulation study 1A: moderate SNP effects in a hit region of moderate LD. Each curve is based on 1,000 simulations. Right plot shows the full ROC curve; left plot shows a zoomed section focusing on the top-scoring SNPs.

**Fig. 5:**
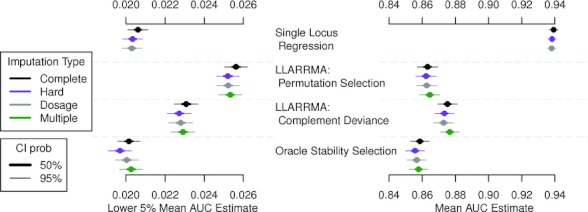
Area under the ROC curve (AUC) for four methods applied to four types of imputed genotype data in simulation study 1A: moderate SNP effects in a hit region of moderate LD. Each AUC estimate is based on 1,000 simulations and is plotted as the mean (dot) with 50% CI (thick bar) and 95% CI (thin bar).

Figure 4 plots ROC curves (see Methods) for each of the four methods, with single locus regression applied to complete genotype data and resample-based methods applied to genotype data with ∼10% missingness that has been multiply imputed (see Methods and below). The ROC curve plots the trade-off between power (the proportion of true signals declared as influential) and FPR (the proportion of background SNPs declared as influential) when thresholding the SNP scores (log*P*s or RMIPs) at different values. The initial ROC is arguably of greater relevance to GWAS than the full ROC because it focuses on enrichment of true signals among the top-scoring SNPs. A method whose top four SNPs are true signals, but which never finds the fifth true signal SNP, is arguably more valuable than one whose top SNPs are false but which finds all five true signals among its middle scoring SNPs. Figure 4 shows both the full ROC curve (right) and the initial ROC curve (i.e., where FPR ≤5%; left). Figure 5 plots the AUC for the initial and full ROC curves for all four methods under four conditions: where the available genotype data are complete, or have ∼10% of genotypes missing but available in hard-, dosage- or multiply-imputed forms. All point estimates (plotted curves in Fig. 4 and mean AUCs in Fig. 5) are based on averages over the 1,000 simulations.

Figure 4 shows, for this example, that single-locus regression most powerfully discriminates true signals from background when the experimenter is prepared to follow up 10% or more of the available SNPs. When at most the top 5% of SNPs can be followed up, however, single-locus regression is dominated by LLARRMA's permutation selection. When follow-up is restricted to the top ∼2% of SNPs, single-locus regression is also dominated by complement deviance and oracle SS. Figure 5 echos these trends. It also shows how the methods perform under different forms of imputation, although no consistent pattern emerges favoring one form over the others.

### SIMULATION STUDY 1B: MODERATE LD, SMALL EFFECTS

**Fig. 6:**
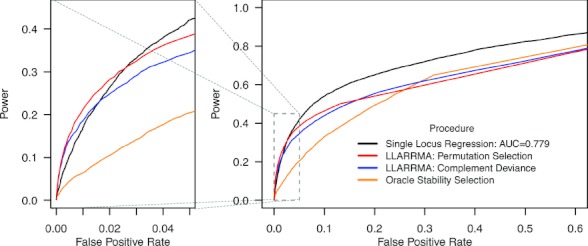
ROC curves for simulation study 1B: small SNP effects in a hit region of moderate LD. Each curve is based on 1,000 simulations.

**Fig. 7:**
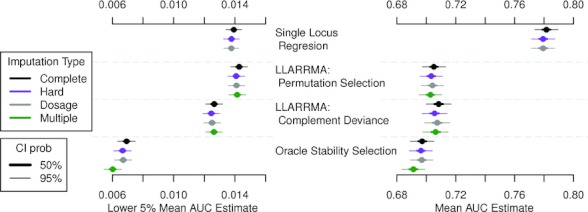
AUCs for four methods applied to four types of imputed genotype data in simulation study 1A: moderate SNP effects in a hit region of moderate LD. Each AUC is estimated from 1,000 simulations.

**Fig. 8:**
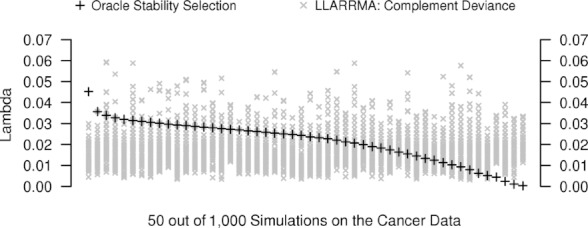
Choice of penalty parameter λ by oracle Stability Selection (black pluses; used to order vertical series along the x-axis) vs. subsample-specific choice by LLARRMA complement deviance selection (gray crosses) in 50 representative simulation trials out of 1,000 performed for simulation study 1B.

We performed a second set of simulations with a design identical to 1A above except with smaller SNP effects (odds ratios around 1.25). The results in Figures 6 and 7 show that although some of the LLARRMA methods dominate in the first third of the initial ROC curve, they generally offer little improvement over single-locus regression under these conditions. The poor performance of oracle SS is striking. “Oracle” refers to the fact that SS was applied in a way that required foreknowledge of the answer: in this case, their free parameter λ was set to maximize their initial AUC. The fact that LLARRMA does not have this oracle advantage and yet still dominates oracle SS suggests a systematic shortcoming of SS in this setting. Figure 8 helps explain the phenomenon. It plots values of the selection parameter λ as estimated by LLARRMA and SS in a representative set of 50 of the 1,000 simulations (using the complement deviance criterion for LLARRMA, which seeks to maximize out-of-sample predictions). Each vertical series shows estimated λ's from one simulation: gray crosses show the 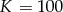 distinct values of λ (i.e., 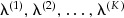) estimated for, and used for selection in, subsamples 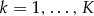 by LLARRMA; black plus signs show the single λ chosen by oracle SS to be applied uniformly across all *K* subsamples. The plot thus contrasts locally optimal vs. globally optimal choices of the selection parameter. In particular, if a dominating strategy allows each subsample *k* to have its own 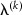 then this plot illustrates how even the best choice of global λ corresponds to a suboptimal local 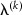 for most subsamples.

### SIMULATION STUDY 2: STRONG LD, SMALL EFFECTS

**Fig. 9:**
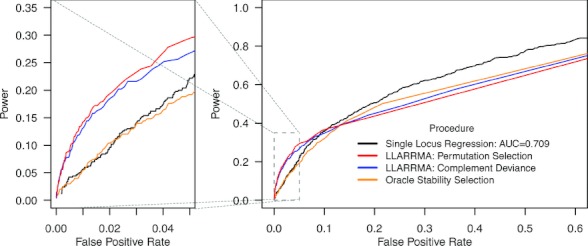
ROC curves for simulation study 2 with 5 true signals: small SNP effects in a hit region of strong LD. Each curve is based on 100 simulations.

**Fig. 10:**
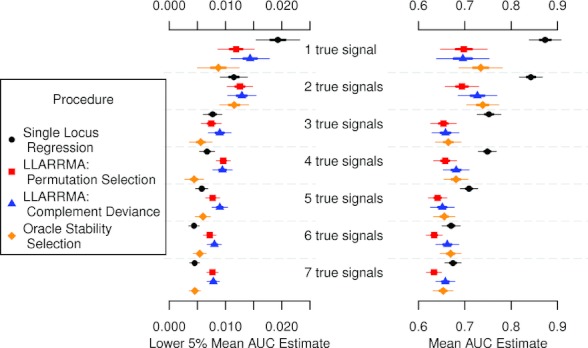
AUC for simulation two with one to seven true signals: small SNP effects in a hit region of strong LD. Each AUC is estimated from 100 simulations.

To examine the relative performance of the single and multiple locus methods in a more challenging setting, we simulated 700 case-control datasets based on the ‘58 data, a region on chromosome 18 containing blocks of strong LD from the GWAS of WTCCC [2007] (see Methods and Fig. 2). Each simulated dataset had a complete set of genotypes and approximately balanced cases and controls. Individuals’ outcomes were influenced by between one and seven true signals of small effect (allelic odds ratios of around 1.25), with 100 simulations devoted to each simulated number of true signals 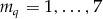. Figure 9 shows the initial and full ROC curves from the 100 simulations in which five true signals were simulated. In this high correlation—weak signal setting, all forms of LLARRMA dominate single-locus regression in the initial ROC curve, suggesting an advantage of simultaneously modeling multiple loci in the presence of high LD. By contrast, oracle SS equals or underperforms single-locus regression, a result similar to that in 1B above, suggesting that this modeling is suboptimal in SS. Figure 10 summarizes results from all 700 simulation trials and shows the effect of varying the number of true signals. With one true signal, single locus regression equals or betters any other method; but as the number of true signals increases, its advantage over multiple locus methods diminishes. In particular, for four or more loci LLARRMA consistently outperforms in the initial AUC, whereas oracle SS consistently underperforms both LLARRMA and single locus regression.

## DISCUSSION

We present a general approach for characterizing frequentist variability in LASSO-based model choice, LLARRMA, and apply it to a problem for which it should be well suited: discriminating true from false signals among a set of SNP predictors that are often highly correlated. In doing so, we evaluate two criteria for automatically choosing the LASSO penalization parameter λ (permutation and complement deviance selection), demonstrate potential superiority of local vs. global regularization in subagging (through comparison with SS), and propose a natural way to combine resampling aggregation with multiple imputation to account more comprehensively for different sources of variability in model choice.

LLARRMA's intended use is in focused analyses on hit regions that have been already identified during whole-genome analysis. Rather than replacing single locus regression, its value lies in what it subsequently adds to that analysis. When there are few causal variants and mild LD, the best LLARRMA methods add little. But when there are many, applying LLARRMA produces a top set of loci that is, on average, enriched for true signals relative to that obtained by single SNP association (cf. ≥4 true loci in simulations 2B). Of our two alternatives for automatically selecting the penalty λ, we found a slight but consistent advantage of permutation selection (modified from Ayers and Cordell [2010]). This could reflect its discovery-based motivation matching our discovery-oriented evaluation, and does not preclude complement deviance, our alternative, being superior in predictive settings.

We explored the use of SS [Meinshausen and Bühlmann, 2010] in this context but find it no better than, and usually inferior to, LLARRMA, despite the fact that our evaluation of SS is based on an optimal calibration of its (unspecified) penalization parameter. One explanation is that SS's use of a single global λ for all subsamples *underfits* the data, in that it fails to accommodate structural differences between LASSO paths fit to different subsamples. The local automatic regularization in LLARRMA implies a different perspective: that λ is a parameter intrinsic to, and only meaningful in the context of, a single LASSO path on a single (subsampled) realization of the data. Another factor could be our evaluation scheme: by calculating power and FPR at different thresholds of RMIP, we (reasonably, in our view) assume that RMIPs should be comparable across simulated datasets. But when we threshold instead on the ranks of the RMIPs within simulated datasets (such that the best RMIP in trial 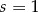 is equivalent to the best RMIP in 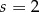), the performance gap between SS and LLARRMA narrows (data not shown), suggesting SS RMIPs are discriminatory but their absolute values are less comparable across studies. Lastly, although our implementation of SS uses subsample proportion 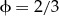 rather than the original 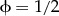 of Meinshausen and Bühlmann [2010], our preliminary studies (not shown) do not suggest this biases comparisons with LLARRMA.

Alexander and Lange [2011] recently demonstrated SS's inferiority to single locus regression for identifying unlinked quantitative trait loci (QTL) in whole-genome association (also using data from WTCCC [2007]). The weakness we identify in SS may help explain that poor performance. Nonetheless, we believe that to expect SS (or LLARRMA for that matter) to beat single locus regression at its own game is not only optimistic, especially given the near optimality of marginal approaches suggested by Fan and Lv [2008], but also distracts from the potential advantages of multipredictor shrinkage for disentangling highly correlated signals in LD blocks following an initial single locus scan.

Multiple imputation is simply accommodated by our resampling scheme, with draws from an arbitrarily complex imputation algorithm dovetailing naturally with the drawing of each subsample from the full data. However, our results suggest that even with 10% missing genotypes multiple-locus inference is served just as well by simpler “plug-in” imputation estimates (hard and dosage). Nonetheless, we advocate multiple imputation where possible because it more comprehensively models imputation uncertainty (among genotypes or other covariates) that could be more pronounced in messier datasets.

Resample aggregation techniques, such as bootstrap aggregation (“bagging”; [Breiman, 1996]) or subsample aggregation (“subagging”; Bühlmann and Yu, 2002]), have been found to produce estimates of 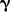 that are more stable than from a single estimation run in the sense that those estimates have lower frequentist risk under squared error loss [Bühlmann and Yu 2002]. We prefer subagging (as in Meinshausen and Bühlmann [2010], Valdar et al. [2009]) to bagging for two reasons. First, theoretical results in Politis et al. [1999, pp. 47–51] suggest that subsampling is less efficient but more general than bootstrapping; specifically, that whereas bootstrap methods must often assume that the estimated statistic is at least locally smooth (which the true or sampled 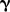 is not), this assumption is not needed for subsampling. Second, resampling individuals with replacement (bootstrapping) poorly approximates variation in GWAS samples because it produces frequent duplicates in a scenario where observing multiple individuals with identical genetic composition would be improbable.

In our simulations, we measure performance by a simple but stringent criterion. We define a “true signal” as the SNP that most strongly tags an underlying causal variant, consider all other SNPs as “background,” and regard success as discrimination of one from the other. In doing so, we provide a criterion for assessment that is unambiguous and in line with many comparable studies [e.g., Alexander and Lange, 2011; Basu et al., 2011; Basu and Pan, 2011; Chun et al., 2011]. Nonetheless, important alternatives exist. A more sophisticated assessment of accuracy, for example, might use a softer criterion that counts as true all SNPs within a given distance or LD-cutoff of a causal locus [e.g., Ayers and Cordell, 2010; Shi et al., 2011; Zhang, 2012]. That more nuanced approach can be helpful in evaluating methods for genome-wide association, but would be counterproductive in our setting for the following reasons. We target hit regions already identified through genome-wide association, and in those regions attempt to discriminate causal from correlative variants in the presence of confounding LD. This goal is not easily reconciled with an assessment mechanism that allows a margin of error in SNP choice. For example, the definition of a useful cutoff for declaring a true positive is highly sensitive to the marker density and the pervasiveness of LD in the hit region of interest, and both of these vary considerably between our two datasets. Moreover, given a suitable cutoff, it is debatable (yet crucial for assessment) whether, for example, one or three hits within range of two causal loci would count as identifying both. Our hard criterion avoids such ambiguities, and provides a stark but clear assessment. It also makes our results particularly relevant to scenarios in which many of the examined variants are essentially indistinguishable, such as for extremely dense genotype or sequence data.

We introduce LLARRMA as an approach for characterizing model uncertainty when working within the frequentist paradigm. Alternative Bayesian variable selection approaches do exist [e.g., Guan and Stephens, 2011; Wilson et al., 2010; Zhang, 2012]. At the request of a reviewer, in Supporting information we assess the performance of a contemporary Bayesian variable selection method (PIMASS; Guan and Stephens, [2011]) within our simulation framework, comparing it with LLARRMA, oracle SS, single-locus regression, and forward selection.

Although we describe LLARRMA in the case-control setting using the logistic model, it is easily extended to the analysis of quantitative traits or any response to which the LASSO can be applied. Similarly, although we model under the simplistic assumption of additive effects and no local epistasis, these assumptions could be relaxed by a more sophisticated specification of locus effects, for example, using the group LASSO [Meier et al., 2008; Yuan and Lin, 2006] or a similar structured penalization scheme.

In summary, we describe an approach for characterizing frequentist variability of model choice in binary data that can be usefully applied to the reprioritization of SNPs in hit regions of a case-control GWAS. The method uses LLARRMA and integrates well with schemes for imputation of missing data. The authors will provide an implementation of LLARRMA an R-package R/llarrma as soon as is practicable.

## Supplementary material

Disclaimer: Supplementary materials have been peer-reviewed but not copyedited.
