# Supplementary material for: Reprioritizing Genetic Associations in Hit Regions Using LASSO-Based Resample Model Averaging
Source: Genet Epidemiol. 2012 Apr 30;36(5):451–62. doi: 10.1002/gepi.21639 (PMC3470705; doi:10.1002/gepi.21639)
Supplement: Supplementary file 1 [file gepi0036-0451-SD1.pdf]

# Supplemental Material for: Reprioritizing Genetic Associations in Hit Regions using LASSO-based Resample Model Averaging

**William Valdar**[1†*], **Jeremy Sabourin**[2†], **Andrew Nobel**[2], **and Christopher C Holmes**[3]

[1] *Department of Genetics, and Lineberger Comprehensive Cancer Center, University of North Carolina at Chapel Hill Chapel Hill, North Carolina, USA*

[2] *Department of Statistics and Operations Research, University of North Carolina at Chapel Hill, Chapel Hill, North Carolina, USA*

[3] *Department of Statistics, Oxford, United Kingdom*

[†] *Denotes equal contribution*

March 21, 2012

## Additional simulation results: forward selection and PIMASS

### Introduction

Herein we report the performance of two additional methods applied to some of our simulations: forward selection terminated by an L0 penalty, and Bayesian variable selection using the PIMASS software of Guan and Stephens (2011). Our assessment is not intended to represent a comprehensive evaluation of those methods: forward selection can be implemented in other ways that may give different results; PIMASS seeks to address a subtly different question and is optimized to work in a more expansive data realm. We expand on this latter point below.

Our main manuscript introduces an approach for characterizing model uncertainty when working within a frequentist framework, and applies it to a task for which it should be ideally matched: the reprioritization of SNP signals in analysis of a hit region. Frequentist literature typically ignores this problem while Bayesian literature explicitly models it. Our manuscript presents a solution for researchers who consider the problem crucially important but prefer not to address it within a Bayesian framework. In short, we condition on being frequentist, and examine the problem of variability or uncertainty of model choice working within that paradigm.

It is nonetheless interesting, however, to consider the performance of a contemporary Bayesian variable selection (BVS) method applied to the same simulations. We note that BVS methods attempt to answer a fundamentally different question, and so a comparison between with LLARRMA, Stability Selection or p-values from single locus regression, will not be strictly meaningful except in the most superficial sense. Specifically, Bayesian approaches model uncertainty about the parameters conditional on the data, whereas the frequentist approach models the expected variability of a statistic (such as an estimate from a model selection procedure) due to variation in different data samples. Because the approaches ask different questions, even optimal implementations of each would not necessarily yield similar answers.

### Methods

All notation follows that in the main manuscript.

*Correspondence to: William Valdar, Department of Genetics, University of North Carolina at Chapel Hill, Chapel Hill, NC 27599-7265, USA. E-mail: william.valdar@unc.edu

**Forward Selection** Forward selection sequentially adds predictors into a model until a predefined stopping criterion is reached. Typically the criterion is based on a trade-off between the fit of the model and a some penalty on the number of predictors included (eg, AIC, BIC). A given penalty thus produces a single binary estimate of $\boldsymbol{\gamma}$ that would correspond to single point in a ROC plot. Providing a full ROC curve requires exploring a continuum of penalties of increasing stringency. However, unlike with the $\lambda$ selection parameter in the LASSO, there is no obvious monotonic function that would accommodate the most popular stopping rules. Therefore, we use a simplistic stopping criterion that is monotonic: a bound on the number of included predictors, $q_{\max}$. Specifically, we identify a subset $q_{\max}$ of candidate SNPs associated with case-control status from $m$ total SNPs using forward selection. Starting with a base model containing only an intercept term, we use binary logistic regression to test the significance of each SNP in turn (as in single locus regression) to identify the most significant SNP. This SNP is then incorporated into the base model and the remaining markers are rescanned to identify the the most significant SNP conditional on the SNP(s) already in the base model. That process is repeated until the model contains $q_{\max}$ SNPs.

**PIMASS** Guan and Stephens (2011) describe a Bayesian variable selection method suitable for QTL identification in genomewide association studies. The method, an Markov chain Monte Carlo sampler implemented in their computer program PIMASS[1], uses a hierarchical prior to model jointly the number of included SNPs and the sizes of their effects. We run PIMASS based on a logit link (option `-cc`) under standard settings, using a 100,000 sample burnin[2] (`-w 100000`) and obtaining results based on 1,000,000 samples (`-s 1000000`). For each SNP, PIMASS provides a Bayes factor (BF) quantifying support for its inclusion in the model.

**Other methods** We considered including BIMBAM (Servin and Stephens, 2007), which not only models multiple loci but also imputation uncertainty. However, we found that applying it to our smallest dataset incurred a severe computational overhead that precluded fair comparison (eg, a single run on the cancer data that allowed up to 3 SNPs to enter the model required 34 hours).

## Results & Discussion

We applied forward selection and PIMASS to data generated by simulation studies 1A (moderate LD, moderate effects), 1B (moderate LD, weak effects), and simulation study 2 (high LD, weak effects) from the substudy that included 5 true signals. The ROC curves and CIs are plotted in Figures 1-6, with single locus regression, LLARRMA and SS included for comparison. The ROC analysis assesses the classification error by thresholding on the level of penalization $q_{\max}$ for forward selection, and on the Bayes factor for individual SNPs for PIMASS.

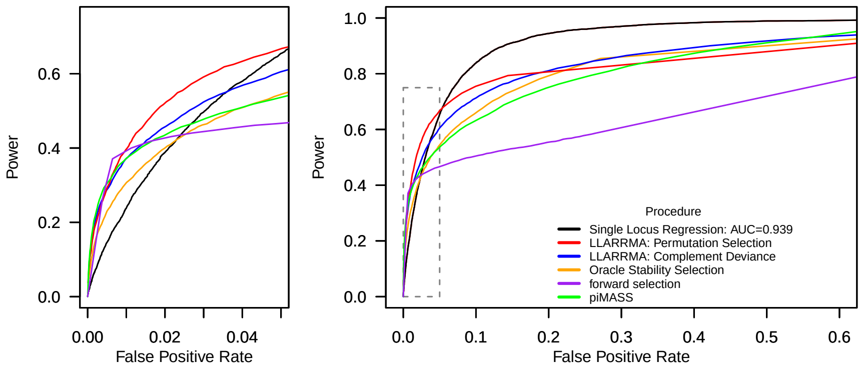


Figure 1: ROC curves for selected methods from simulation 1A with the complete cancer data.

The results from the cancer data (simulations 1A and 1B; Figures 1-4) show that both forward selection and PIMASS perform similarly to LLARRMA at the very start of the ROC but quickly deteriorate thereafter.

[1]http://www.bcm.edu/cnrc/mcmcmc/pimass
[2]Experimenting with a longer burnin of 1,000,000 gave similar results, suggesting that 100,000 is adequate.

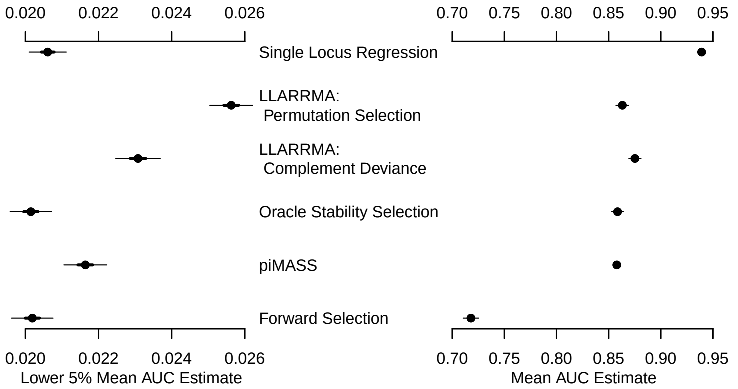


Figure 2: Area under the ROC curve for selected methods from simulation 1A.

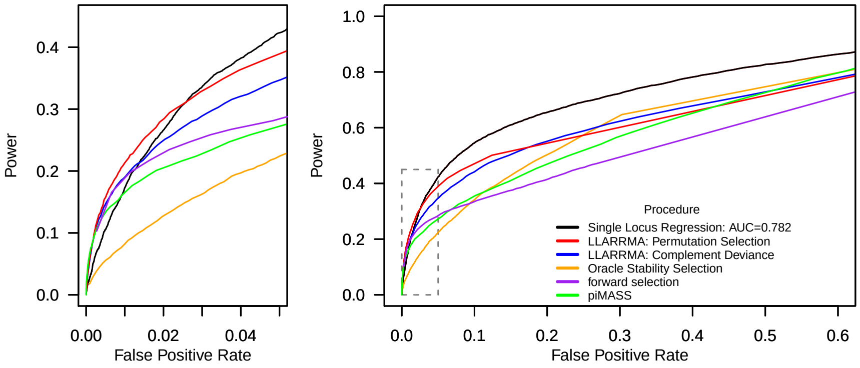


Figure 3: ROC curves for selected methods from simulation 1B with the complete cancer data

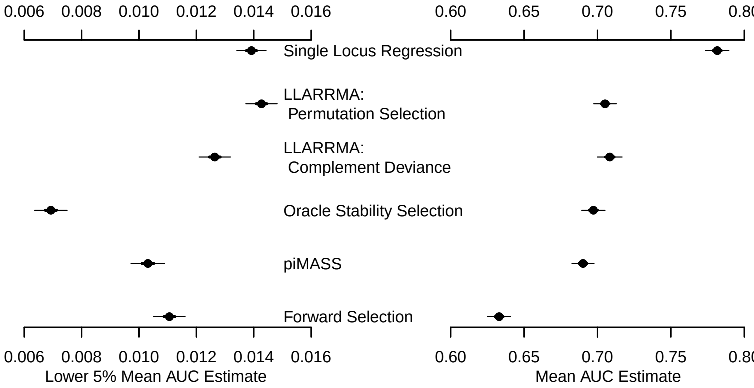


Figure 4: Area under the ROC curve for selected methods from simulation 1B.

The results from the 58 data suggest that PIMASS and forward selection perform especially poorly in hit regions of high LD. This is unsurprising for forward selection, which contains no mechanism (such as penalization or hierarchical priors) for splitting effects across correlated loci. For PIMASS, which does, we speculate that underperformance results more from a poor match of its default MCMC settings to the high correlation in this hit region. Specifically, PIMASS implements swap proposals for spreading probability over neighboring SNPs (Guan and Stephens, 2011). This should produce an effect analogous to that motivating, eg, a randomized LASSO (Meinshausen and Bühlmann, 2010). However, we suspect that PIMASS's current implementation might assume that LD decays more quickly than in our high LD hit region.

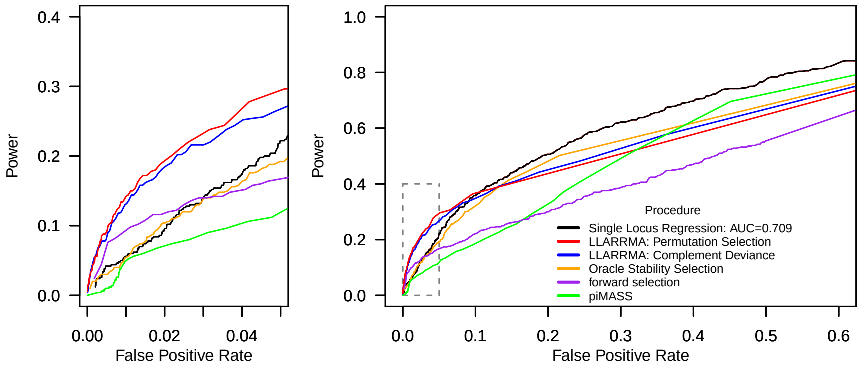


Figure 5: ROC curves for selected methods from simulation 2 with 5 loci the '58 data.

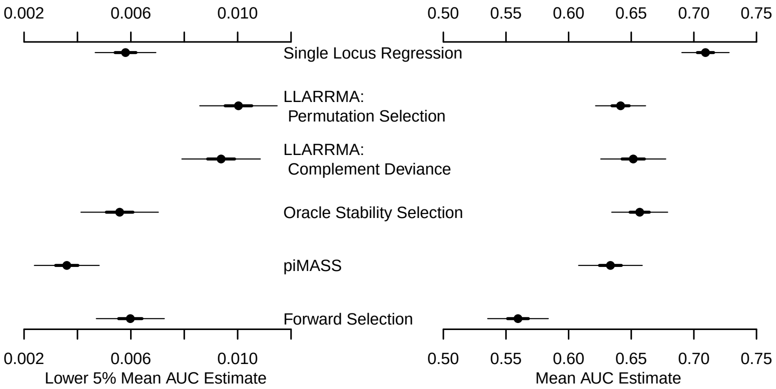


Figure 6: Area under the ROC curve for selected methods from simulation 2 with 5 true signals.

# References

Guan Y, Stephens M. 2011. Bayesian variable selection regression for genome-wide association studies, and other large-scale problems. Annals of Applied Statistics 5:1780–1815.

Meinshausen N, Bühlmann P. 2010. Stability selection. J Roy Stat Soc B 72:417–473.

Servin B, Stephens M. 2007. Imputation-based analysis of association studies: candidate regions and quantitative traits. PLoS Genetics 3:1296–1308.
